# Barriers and Facilitators for Conducting Implementation Science in German-Speaking Countries: Findings from the Promote ImpSci Interview Study

**DOI:** 10.1007/s43477-022-00046-3

**Published:** 2022-05-26

**Authors:** Marie-Therese Schultes, Monika Finsterwald, Thekla Brunkert, Christina Kien, Lisa Pfadenhauer, Bianca Albers

**Affiliations:** 1 Institute for Implementation Science in Health Care, University of Zurich, Universitaetstrasse 84, 8006 Zurich, Switzerland; 2 Department of Developmental and Educational Psychology, University of Vienna, Vienna, Austria; 3 Nursing Science (INS), Department Public Health, University of Basel, Basel, Switzerland; 4 University Department of Geriatric Medicine FELIX PLATTER, Basel, Switzerland; 5 Department for Evidence-Based Medicine and Evaluation, Danube University Krems, Krems an der Donau, Austria; 6 Chair of Public Health and Health Services Research, Department of Medical Informatics, Biometry and Epidemiology (IBE), LMU Munich, Munich, Germany; 7 Pettenkofer School of Public Health, Munich, Germany

**Keywords:** Implementation science, Capacity building, Research infrastructure, Training, Scientific networks, Research funding

## Abstract

On a global scale, implementation science has developed considerably as a discipline in recent years. In German-speaking countries, the field has been gaining significance as well, but respective efforts in building capacity and advancing the research infrastructure are still rare. The present study investigates barriers and facilitators for conducting implementation science in German-speaking countries with the goal of formulating recommendations for creating a more supportive research infrastructure. We conducted an interview study with nine well-established implementation researchers affiliated with universities in Austria, Germany, or Switzerland. The interviews were held via Zoom or phone in November and December 2020, transcribed verbatim and analyzed using thematic analysis. Barriers that relate to characteristics of the discipline were difficulties in building a common understanding of the field and the complexity of implementation research projects. Although supportive scientific networks were important facilitators, interviewees mentioned challenges in connecting with likeminded researchers. A further barrier was the lack of opportunities for education and training in implementation science, especially in the German language. Also, participants reported a missing readiness in academia for establishing implementation science that should be addressed by advocacy of the discipline toward academic decision makers. Moreover, since most national funding agencies prioritize basic research over applied research, some interviewees named flexibility in handling research funds as a facilitator for implementation research. The results inform an agenda for promoting implementation science in German-speaking countries and can be beneficial to other countries that are currently advancing their implementation research capacity and infrastructure.

**Supplementary Information:**

The online version contains supplementary material available at 10.1007/s43477-022-00046-3.

Time lags in the research translation process have been an issue of debate for a long time (Hanney et al., 2020; Morris et al., 2011). At their core has been a concern about considerable delays with which research has been translated and used in routine practice. Implementation science offers evidence-based strategies on how to close the research to practice gap in a variety of fields, such as health, education, and social welfare (Albers et al., 2020). However, establishing implementation science requires a paradigm shift from traditional research infrastructures that commonly focus on clinical or basic research to the application of scientific knowledge in practice.

Implementation scientists operate on the basis of theories, frameworks and models (see, e.g., Nilsen, 2015) that are continuously advanced and inform implementation projects in diverse practice settings. Implementation research projects are often conducted in interdisciplinary teams, include the perspectives of different stakeholder groups, and consider various system levels at once (Schultes et al., 2019). If the goal is to sustainably implement an innovation into a complex practice setting, implementation science projects need appropriate resources and time. Accordingly, implementation research requires a research infrastructure that supports complex, long-term research.

Implementation science has grown rapidly in the last two decades (Albers et al., 2020), while frequently attending to current research transfer problems. For example, with regard to substantial public health challenges such as the SARS-CoV-19 pandemic, implementation science offers knowledge on the long-term implementation of preventive measures as well as the reduction of adverse side effects caused by these measures (Wensing et al., 2020). Moreover, implementation scientists propose strategies for building national and international public health capacity (Aijaz et al., 2021) and help to better understand the role and relevance of different implementation contexts in these efforts (Pfadenhauer et al., 2017), highlighting that implementation research needs to consider the socio-cultural, socio-economic, and political circumstances (Shelton et al., 2020). Hence, to make use of implementation science in a geographic region, it is crucial to both study the results of international research and to build capacity for implementation research and practice in the respective region.

To date, research activities, funding opportunities, and formal education in implementation science have been concentrated on certain geographic regions, especially the United States of America, Canada, the United Kingdom, and Northern Europe (see, e.g., Baumann et al., 2020; Davis & D’Lima, 2020). In contrast, most Western European countries, including German-speaking countries, are just getting started in both using and advancing implementation science (see, e.g., Hoben et al., 2014). To establish implementation science as a scientific discipline, it is crucial to know supportive and inhibitory factors for conducting this kind of research.

## Barriers and Facilitators for Conducting Implementation Science—An International Perspective

In the following, we summarize findings regarding barriers and facilitators that have shown to influence the conduct of implementation science in other countries and regions. The present study was developed using this global literature, which we revisit in the discussion of our results from German-speaking contexts.

US-based health researchers have described the increasing visibility and perceived relevance of implementation science as a facilitator for engaging in the field (Stevens et al., 2020b). Moreover, being exposed to implementation science through educational materials or introduced to the discipline by mentors or colleagues were reported as motivating factors (Stevens et al., 2020b). Similarly, support by the research community and by researchers in leadership positions have been positively related to an engagement in implementation science (Stevens, 2021). However, researchers also have described it as challenging to form collaborations with other implementation researchers and community partners (Stevens et al., 2020b). In a concept mapping study on international collaborations in implementation research, participants rated joint funding as one of the most important, though not very feasible, facilitators (Aarons et al., 2019). In some countries, such as Australia, Canada, and Ireland, there is an increase in funding streams that support implementation science (Koorts et al., 2020), while implementation research funding opportunities are scarce in other countries, including German-speaking countries.

Further challenges for engaging in the field have been related to the difficulty of defining implementation science, especially in contrast to other research disciplines (Stevens et al., 2020b). Moreover, the prioritization of efficacy studies in most academic environments has been described as a major obstacle for engaging in the field (Koorts et al., 2020). Correspondingly, researchers have expressed concerns about having difficulties with publishing results from demanding implementation research projects. This has been reported as a problem especially for early career researchers with limited time, for example, to finish their PhD publications (Koorts et al., 2020). Accordingly, those who saw potential disadvantages for their career development refrained from engaging in implementation science (Stevens et al., 2020a).

Concerning education in implementation science, prior research has shown that many relevant competences for implementation research are acquired by self-study (Schultes et al., 2021). There is a growing number of webinars, workshops as well as courses and modules in academic curricula dedicated to capacity building in implementation science (Davis & D’Lima, 2020). These courses and training programs not only serve educational purposes, but also provide implementation researchers with valuable networking opportunities (Davis et al., 2020). However, in general there is a higher demand than supply of implementation training, which becomes apparent in highly selective eligibility criteria and low acceptance rates in training initiatives (Davis & D’Lima, 2020). Moreover, the lack of training opportunities has been reported as a barrier for engaging in implementation science (Koorts et al., 2020; Stevens et al., 2020b).

Internationally, there is an increasing number of practice examples in how to strengthen facilitators and overcome barriers for engaging in implementation research. For example, in presenting the Washington University Network for Dissemination and Implementation Research (WUNDIR), Brownson et al. (2017) describe how to build support structures at the university level. For establishing institutional support for implementation science, essential factors include promoting a transdisciplinary dialogue by facilitating meetings across departments and building networks with other universities and community partners (Brownson et al., 2017). Transdisciplinary exchanges in implementation research also are fostered by the increasing number of international implementation science conferences available to researchers (e.g., Bawab et al., 2020; Landes et al., 2020). Moreover, there is a multitude of national and international implementation science networks (Shelton et al., 2020). Most of these are interdisciplinary networks and include a wide variety of research fields, while some specialize in implementation research with a particular focus, such as adolescent HIV prevention and treatment (Sturke et al., 2020) or capacity building in cancer prevention and control (Friedman et al., 2021).

## Implementation Science in German-Speaking Countries

In German-speaking countries, mostly isolated efforts have been made thus far to build a comprehensive implementation science infrastructure. For example, the University of Heidelberg, Germany, offers a Master of Science (MSc) program in Health Services Research and Implementation Science (Ullrich et al., 2017). Implementation science also has been included in curricula in the form of single courses (e.g., in the MSc psychology at the University of Vienna, Austria, the MSc nursing science at the University of Basel, Switzerland, or in the MSc health economics and health care management at the Bergische Universität Wuppertal, Germany). However, even though opportunities for education in implementation science are growing, they mostly target specific groups from single disciplines. Moreover, only few higher education institutions have designated chairs for implementation science.

The rising interest in the field also is reflected in the growing community of the German-Speaking Implementation Association, an informal network of researchers, practitioners, and policy makers with an interest in implementation science that was founded in 2017. The network communicates via social media and newsletters and connects people with special interests, for example, in teaching implementation science. Furthermore, the Swiss Implementation Science Network IMPACT has been founded with the goal of increasing awareness of the field in Switzerland and internationally (Dhaini et al., 2021).

Beyond education and networks, there are not many support structures for implementation researchers available in German-speaking countries. To our knowledge, there are no designated funding streams and journals or initiatives that support early career researchers in implementation science. Moreover, researchers from different disciplines use various labels when translating implementation science to German, which makes it difficult to grasp the current state of the field. Addressing this problem, recent translations of central implementation science concepts to German (Gutt et al., 2018; Regauer et al., 2021) provide an important basis for a common language. Still, there are only a few publications about implementation science in German (e.g., Baumeister, 2014; Beelmann & Karing, 2014; Schober et al., 2019), and there are hardly any German language resources for educational purposes. With implementation science being an applied field, this makes it difficult to recommend literature to project partners or practitioners in continuing education who are not used to working with resources in the English language.

## Present Study

As a reaction to the missing implementation research infrastructure, the first author initiated the research project “Promote ImpSci—How to support implementation science in German-speaking countries”. So far, the project activities included an interview study and a workshop with the German-speaking implementation science community. This paper reports on the results of the interview study, which was conducted in winter 2020. The objective of the present study was to investigate barriers and facilitators for conducting implementation science in German-speaking countries. We studied the facilitators and resources that are already available to researchers and discussed support structures that should be developed or strengthened to create a supportive implementation research infrastructure. Since, to our knowledge, there has been no previous research on this topic in the respective context, we chose to follow an inductive qualitative approach.

## Methods

The presentation of our methods follows the SRQR reporting guidelines (O’Brien et al., 2014, see Supplementary information 1). We conducted semi-structured expert interviews with a purposive sample of well-established implementation researchers in German-speaking countries. The expert interviews did not require formal review by an ethics review board, since the study did not involve patients, was non-invasive and participation was voluntary and anonymous. Due to limited funding, we had to restrict our sample to nine interviewees. Selection criteria included an affiliation with a higher education institution in Austria, Germany, or Switzerland and visibility in the implementation research community through publications or scientific networks. An institution could only be represented by one implementation researcher in the sample. Potential interviewees were contacted via email, and, after having accepted the invitation, were sent a list of interview topics (see Supplementary information 2). The topics were developed by the first author on the basis of prior research (Stevens et al., 2020b) and reviewed by all co-authors. The interview guide included questions on the interviewees’ personal implementation research experiences, barriers and facilitators for conducting implementation science in German-speaking countries, resources and activities for German-speaking implementation researchers and demographic data. We did not change the interview guide over the course of the study.

The first and last author (MTS & BA) held the interviews via Zoom or phone in November and December 2020. Eight interviews were held via Zoom and one interview was held via phone, due to the interviewee traveling during the interview. The interviews conducted via Zoom had a better audio quality and the advantage of the interviewers being able to react to nonverbal cues during the discussions. Eight interviews were held in German and one interview was held in English. We informed the participants about the goals of the study and the study procedure, including their data being anonymized. After gaining the interviewees’ informed consent, interviews were recorded with an audio recorder and then transcribed verbatim. The English interview transcript was not translated to German for data analysis. The audio files were deleted after the transcripts were produced. Transcripts were sent back to the participants, who had the opportunity to edit potential misinterpretations. This opportunity was used by five participants, who mostly proposed minimal edits. Then, transcripts were anonymized. A group of three researchers, who are highly experienced in qualitative research (MTS, MF, BA), analyzed the data using Dedoose for an initial data screening and MAXQDA 2020 for detailed coding. Our data analysis followed an inductive approach and was guided by the steps of thematic analysis (Braun & Clarke, 2012). We chose this approach mainly because of its flexibility with handling data of a relatively new field of study.

First, all three researchers familiarized themselves with the data by reading all transcripts and producing memos on potential themes and codes. After discussing the memos in the group, one researcher (MTS) created an initial coding system that was tested independently by two researchers (MTS & MF) using the same two transcripts. Experiences with this step and coding discrepancies between both researchers were discussed in the whole coding group. As a result, the coding system was revised, and all nine interviews were double coded in dyads. The dyads gained average intercoder agreements of *k* = 0.77 and *k* = 0.79, respectively, and again, discussed their experiences and discrepancies. As a result, the final codes were named and grouped into themes by one researcher (MTS). All three researchers discussed and revised the themes, which formed the basis of our results.

## Results

### Participants

Nine implementation researchers participated in the interview study. The interviewers knew four of the participants personally prior to conducting the interviews. The interview duration ranged between 25 and 60 min. Five participants were affiliated with higher education institutions in Germany, two with institutions in Austria and two with institutions in Switzerland. Six participants were full professors at the time of the study, with the other three being senior pre- (*n* = 1) and postdoctoral researchers (*n* = 2). Their experience with implementation science ranged from five to 28 years (Median = 10 years). Most interviewees worked in health sciences (*n* = 6), followed by educational and prevention research (*n* = 2) and social work science (*n* = 1).

### Barriers and Facilitators for Conducting Implementation Science in German-Speaking countries

We derived a total of seven themes from the thematic analysis that describe barriers and facilitators for conducting implementation science in German-speaking countries. Each theme comprises between two and six codes. The final coding system, including sample quotes for each code, can be found in Supplementary information 3. The themes, which we describe in detail in the following, include: characteristics of implementation science as a scientific discipline, common conditions of implementation research projects, personal factors that relate to conducting implementation research, linkages with and networking among implementation scientists, possibilities for acquiring implementation science competences, readiness in academia for establishing implementation science, and funding implementation science.

#### Theme 1: Characteristics of Implementation Science as a Scientific Discipline

Most interview partners described implementation science as a rather new, emerging field in their country. The early developmental stage of the field contributes to certain barriers for conducting implementation science. One barrier is the low visibility of implementation science in public and policy. As an example, one interviewee named the absence of implementation science expertise in the development and implementation of preventive measures concerning the SARS-CoV-2 pandemic. Another challenging characteristic is the lack of a common understanding of what can be described as implementation science. Several interview partners described the different notions of what constitutes implementation science in different facets. One facet was its differentiation from applied research in general:

I have the impression that a lot of things are understood as implementation science. There are examples of people, who think they conduct implementation science, because what they do is practice related. So, implementation science seems to be practice-related research to some people. (…) I think there is no common understanding about what implementation science is, at least not in Germany. For example, I ask myself if it is part of health services research or not (I1).

Other interviewees mentioned that they have observed colleagues using the term implementation science for their research without being familiar with theoretical and conceptual basics of the field, such as implementation frameworks and models. One interviewee viewed this as an expression of implementation science being a buzz word in some disciplines that is just used to gain recognition in the respective scientific community. Another interviewee observed that, in comparison to countries where the field is more established, there are hardly any scientists in German-speaking countries who focus solely on implementation research. In general, almost all interviewees shared the impression that implementation science is more advanced in other countries, predominantly naming the USA as an example.

Participants described two characteristics of implementation science that can be categorized as both barriers and facilitators, namely its applied nature and interdisciplinarity. One interviewee appreciated that the involvement of stakeholders leads to an engagement in practically relevant research that benefits the return on investment in research for society. At the same time, the conditions of working closely aligned with practice are not always compatible with everyday academic work:

You cannot just sit in a university and do implementation science from your desk. You need a field where you can really study it and learn. This applied aspect makes it difficult to separate clinical from academic aspects (I3).

Some interviewees saw the interdisciplinary nature of implementation science as an advantage since it allowed them to get to know and even work in different fields. Others talked about difficulties of developing a common profile for the discipline when researchers from different disciplines use different labels for their implementation research activities. For example, several interviewees mentioned the challenge of distinguishing implementation science from health services research (“Versorgungsforschung” in German), since implementation science is often seen as a subdiscipline of health services research in German-speaking countries.

#### Theme 2: Common Conditions of Implementation Research Projects

Most participants pointed to the large investment in time and effort needed for implementation research projects as an important barrier to engaging in the field. It was described as especially difficult for researchers working on temporary contracts to invest themselves in and be able to finish long-term implementation projects. This was explained as being related to high costs of most implementation research projects:

I know from the US how expensive, how difficult, how involved implementation research is and I don’t see that there is money available for that (I7).

Implementation research projects usually are conducted in collaboration with external partner organizations. Most participants viewed this condition as challenging in the context of academia, since building constructive long-term collaborations with community partners is time-consuming and often requires a strategic approach that many researchers do not feel well-equipped for:

It is extremely difficult to get resources, and therefore you need partners from practice and administration, who organize that and who have a better connection to policy makers, compared to scientists. (…) I couldn’t do it, I’m not a politician who negotiates things and acts strategically and thinks about how things look to the public. For that, you need people who do that and who are able to do that (I9).

#### Theme 3: Personal Factors That Relate to Conducting Implementation Research

When talking about their personal motivation for engaging in implementation science, most interviewees mentioned that they had observed a lack of use or transfer of evidence-based knowledge in their respective practice settings. More concretely, several participants mentioned frustrations with elaborately developed interventions not being implemented in routine practice:

(…) what I found so frustrating in my early research (…), that something was developed and largely evaluated, but then no one really did anything with it anymore (I6).

A supportive factor for engaging in implementation research was the interviewees’ own determination to enable this missing transfer. Hence, some participants mentioned their personal drive to identify possibilities for launching even small implementation science projects and for starting research collaborations. These were often found at international implementation science training initiatives and while working on research fellowships abroad that facilitated the collaboration with other implementation scientists.

#### Theme 4: Linkages with and Networking Among Implementation Scientists

Almost all interviewees stressed the importance of international networks for engaging in and conducting implementation science and emphasized the role of the international partners or mentors who had introduced them to the field. Additionally, supportive mentors who are part of the interviewees’ own research group were highlighted as an important facilitator:

(…) there is great support in our department because our management thinks it is important. If that would not be the case, someone like me would not be here and would not get any resources. That depends very much on specific people (I3).

Moreover, having other people in the research team or department who are interested in implementation science was named as a facilitator. However, in thinking beyond the participants’ own research institution, several interviewees described it as difficult to find collaboration partners in their country who work on similar research topics. According to some participants, this is due to difficulties in building an interdisciplinary network in the German-speaking academic landscape. Most conferences and journals address specific disciplines and cross-pollination between these disciplines is limited. One interviewee described similar studies being conducted simultaneously and in the same country because research groups do not know of each other. Another problem being discussed was finding collaborations to strengthen implementation research proposals:

(…) if I would write an implementation science research proposal in [a German-speaking country], I would have to think hard about whom to include as a collaboration partner (I6).

#### Theme 5: Possibilities for Acquiring Implementation Science Competences

Several interviewees pointed to the scarcity of formal implementation science education and training opportunities in German-speaking countries as a barrier to engaging in the field. Making implementation science a part of more academic curricula was described as an opportunity to gain higher visibility for the field, especially among early career scientists:

I think that implementation science is not a topic (in academic curricula) and because of that it is not known. (…) It would be good to get young people’s attention and to give them the opportunity of specific education and training (I2).

Moreover, some participants saw it as essential to provide implementation science training opportunities in the German language to overcome language barriers. Those interviewees, who had participated in formal training, had done so abroad. Here, one participant named their access to travel funds for international training opportunities an essential facilitator. However, most participants had acquired their implementation science competences in self-study, which was described as challenging by one interview partner:

(…) I think it is quite difficult [to decide] where to begin – there is a variety of models and papers and frameworks and to really get into it – do I really have an overview? Sometimes I am not sure, if I got it right, even though I’ve been working on it for years and try to read a lot (I5).

#### Theme 6: Readiness in Academia for Establishing Implementation Science

Most interviewees shared the impression that there is no general awareness of implementation science in German-speaking academia. One participant shared their experience that other academics are often surprised about implementation being a field of scientific inquiry. Moreover, several participants stated that the problem goes further for some disciplines that do not fully accept the concept of evidence-based practice, at least in their tradition in German-speaking countries:

(…) there needs to be the assumption that evidence-based practice is a good idea. That it is possible to conduct studies that lead to results – not just one study but all studies together – that are the basis for recommendations or directions for practice or policy (I1).

One interviewee also talked about challenges in the prevailing publishing culture of some research teams that demands publishing in discipline-specific journals as opposed to implementation science journals. However, a facilitator that was mentioned several times concerned the growing number of implementation science journals, although some participants named the absence of publications in the German language as a barrier to the dissemination of implementation science.

Along with these challenges in the academic culture, participants also described structural barriers, such as the lack of academic positions dedicated to implementation science. Several participants formulated the goal that there should be more implementation science advocacy directed at stakeholders in academia. More specifically, one interviewee mentioned the importance of spokesmanship to get beyond mere intervention development:

Maybe we need to do more lobbying [for implementation science]. To point out how important it really is. I don’t know (…) if it is known how long it takes for evidence-based interventions to come to practice, some of them never make it to practice and for many we know – I know a study that speaks about 17 years, and I think there are many more that have determined similar timeframes. I don’t know whether this is known among decision makers. Because then they wouldn’t just fund [intervention] development, development, development (I2).

#### Theme 7: Funding Implementation Science

Most interviewees stated that it is difficult to receive funding for implementation research projects by national funding agencies, which prioritize basic over applied research. This can be problematic since project funds by these agencies are seen as the most prestigious and valuable for career advancement:

Ultimately, [project funding by a national funding agency] is the standard, that is what you hope to get. And if this is not possible, if implementation science is not funded for my discipline by [this agency] (…), if a proposal would be reviewed by traditional educational scientists, I don’t see any chance that such a project would be funded. I haven’t tried it, but that is my impression. I think that this is different if you work in health sciences (…) (I7).

However, getting funds from a national agency was less challenging for participants working in health sciences, who stated that they had been able in the past to secure funds for implementation research projects. Several interviewees named European funding schemes, such as Horizon Europe (European Commission, 2022), as a promising way to finance implementation research projects, since respective calls value both considerations on implementation and including stakeholders in the scientific process. Here, several interviewees talked about the practice of using implementation science at least for secondary research questions, while one participant criticized that implementation science is often seen as accompanying research, which they considered as not being helpful for advancing the field.

A certain flexibility in using research funds was named by several interviewees as a helpful facilitator for conducting implementation science, since it enabled them to set their own research agenda. This applies both to the launch of research projects that do not fit into traditional funding schemes and a flexible use of travel funds that allow for attending implementation science training abroad.

## Discussion

The aim of the present interview study was to investigate barriers and facilitators for conducting implementation science in German-speaking countries. Expanding previous research, the present study was the first to investigate preconditions for conducting implementation science in Central Europe and the first to include the perspectives of various disciplines. In an interview study with nine well-established implementation researchers in Germany, Austria, and Switzerland, we explored barriers and facilitators for conducting implementation research. The themes we identified relate to implementation science as a discipline and corresponding research projects, implementation researchers, their networks, and acquisition of competences, as well as the academic and funding system. Most findings mirror prior international studies on factors that relate to engaging in implementation research, while some are especially relevant to German-speaking countries.

Concerning implementation science as a scientific discipline, previous studies (Koorts et al., 2020; Stevens et al., 2020b) have reported its increasing visibility as a facilitator for engaging in the field. In contrast, our participants saw the missing visibility of implementation science in German-speaking countries as a barrier for the establishment of the field. Moreover, the low visibility leads to missing opportunities for including implementation expertise in the public discourse, e.g., concerning public health measures targeting the SARS-CoV-2 pandemic.

A similarity to other studies was the difficulty of determining what implementation science stands for. In our analysis, the code “distinguishing implementation science” was even the most prominent one. According to our interviews, this issue has two facets: First, the challenge of differentiating implementation science from other disciplines, such as health services research, and second, observing other researchers using the term implementation science as a label for their work, even though they are not familiar with its theoretical basics. Further discussions could center around whether this issue can be attributed to implementation science being a comparatively new field and with the word “implementation” being used in the everyday discourse without many people being aware of the field’s scientific foundations. Furthermore, it would be insightful to explore how the term “implementation science” is understood in other countries with different languages and different academic traditions.

The growing number of international interdisciplinary conferences contributes to a common understanding of and language in implementation science, yet events that especially address the German-speaking implementation research community have been scarce. A virtual workshop held as part of the Promote ImpSci project was attended by over 50 German-speaking implementation researchers and practitioners. It was positively received by attendees as the first opportunity for many to meet other implementation researchers from a similar geographic context. This created a space for discussing context-specific topics, such as instrumentation issues that occur when translating implementation outcome measures into German (see, e.g., Kien et al., 2021). At the end of the workshop, participants formulated an agenda for establishing implementation science in German-speaking countries. Similar to the results of the present study, they argued for producing more resources in the German language, creating more possibilities for exchange between implementation researchers and increasing the visibility of implementation science through diverse media outlets. Moreover, the workshop led to a continuous exchange between attendees on topics of special interest, such as implementation science education in the German language.

To successfully conduct implementation research projects, researchers often have to build long-term collaborations with community partners. However, our results suggest that for researchers, these are hard to initiate and maintain, especially when being highly involved in academic daily business. Here, special research transfer units at universities that facilitate communication and collaboration between university members and community partners can be highly supportive. Building such support structures reflects the degree to which universities value their researchers’ work on creating societal impact and could create synergies between different disciplines.

Moreover, the long duration of implementation science projects makes it difficult for academics with temporary contracts to engage in the field. This aspect is particularly challenging for early career researchers. This problem was also reported by Koorts et al. (2020) for the international research community in the field of nutrition and physical activity. In Austria and Germany, novel developments in contract terms for early career scientists limit the possible duration of working on temporary contracts. These amendments put enormous pressure on early career scientists to exceed in academic assessment criteria, such as reaching a high number of publications, in a very short time. Hence, building relationships with community partners and working on long-term projects that have potential sustainable impact are not encouraged by most academic working environments, especially at an early career stage.

The issue that researchers are concerned about engaging in implementation science not being advantageous for their career has been reported previously (Stevens et al., 2020a). This is emphasized by our finding that participants doubt the fundability of implementation research projects by prestigious national funding agencies. This indicates that implementation science is not yet considered a priority in research agenda setting and that the field’s added value still needs to be justified in German-speaking countries, which does not motivate young researchers to move into the field. In contrast, an important facilitator was researchers making use of rare opportunities, for example, for launching even small implementation science projects. In addition to this intrinsically motivated efforts, supportive group leaders play an important role for researchers who decide to follow this career path (Stevens, 2021). Several of our interviewees deemed mentorship a highly relevant facilitator, both in terms of local supervisors and international collaboration partners who have introduced them to the field.

Despite the growing number of implementation science networks, our results support prior findings that many implementation scientists find it challenging to build collaborations with likeminded researchers, especially from other disciplines. To support transdisciplinary collaborations, networks such as the German-Speaking Implementation Association need to be strengthened, for example by making them available through diverse communication channels and by tailoring activities to the members’ needs. At the same time, national networks need to connect to international networks to foster international contacts, which are, according to our participants, necessary for being able to engage in the field.

Training initiatives provide good opportunities for connecting with peers. However, similar to previous research (Koorts et al., 2020; Stevens et al., 2020b), our results showed that researchers feel that there are not enough education and training opportunities in implementation science, especially in the German language. Developing courses for beginners creates possibilities to foster a common understanding of and a shared language in implementation science. For this purpose, exchanging ideas with other educators and attending to a certain consistency in training is helpful (Stevens et al., 2020b). This can be challenging when designing training programs for a diverse, transdisciplinary audience (Davis et al., 2020). Here, it can be beneficial to engage multidisciplinary training staff, for example, for covering different modules in a training program. Moreover, it is important to define the competences that trainees should acquire in a course or program. A competence profile that can guide education of implementation researchers and practitioners has already been developed and can be advanced on the basis of educators’ experiences (Schultes et al., 2021).

In terms of barriers located at the academic system level, interviewees particularly mentioned difficulties in getting funding for implementation research projects. In contrast to other countries, where the field has a longer tradition, there are only few funding streams where such projects would fit the respective scope (for example, the National Research Programme "Smarter Health Care" of the Swiss National Science Foundation). Receiving funding was reported to be even more difficult for projects in education and social work than for projects targeting topics in health care. Here, it is difficult to determine whether the problem concerns implementation science in particular or receiving funds for applied research in these fields in general.

However, the issue that it is easier to receive funding for the development of interventions than for the systematic implementation of existing effective interventions applies to many disciplines and has been observed internationally (Stevens et al., 2020b). A certain flexibility in using research funds was a reported facilitator, which may also be helpful for handling the unpredictability that comes with studying complex implementation processes in real-world settings. The missing readiness in academia for establishing implementation science as a distinct discipline should be addressed through substantial advocacy such that academic decision makers, for example in funding agencies, get to know the field and understand its importance.

The present study is the first investigation of barriers and facilitators for conducting implementation science in German-speaking countries. Hence, a strength of the present study is that it can inform the building of implementation science capacity in countries that are similarly just beginning to establish implementation science as a discipline. Overall, our findings highlight the importance of the infrastructure available to build such a discipline—in the form of education and training, academic roles, research funding, and publication opportunities. It is of additional importance to examine how to best differentiate implementation science from other disciplines attending to similar research questions in a region or country, such as health services research in German-speaking countries. The results of our study might particularly appeal to groups who work on building capacity for implementation research and practice in other languages than English. For this intention, developing resources in the local language, especially for education and training, but also for collaboration with practice partners, is a particularly important facilitator.

## Limitations and Future Research

A strength of our interview study is the inclusion of participants with substantial experience in both implementation science and the higher education system in German-speaking countries. As a result, we could gain valuable information despite the small sample size. At the same time, due to the small sample, our study might not be generalizable to the whole German-speaking implementation community. In particular, our study lacks the diverse perspectives of implementation researchers whose work is not as visible to the scientific community and who might be able to provide more information on barriers for engaging in the field. Further research also could include the perspective of funders and their impressions of whether implementation research questions have gained relevance in recent years.

Moreover, further research on the topic should include more early career researchers whose perspectives could give insight into future developments of the discipline. Also, it should be investigated how the predominantly precarious work conditions of early career researchers relate to difficulties for engaging in implementation science and how their situation differs from other disciplines. Similar studies in countries that are establishing implementation science in other languages than English could furthermore add to the generalizability of implications for building a supportive implementation science infrastructure from a global perspective.

Finally, since the authors themselves are implementation researchers working in German-speaking countries, the potential influence of their own experiences and insights on their interpretation of study results needs to be considered. Also, the authors knew four of the nine participants personally prior to conducting the interviews from their own research networks. To gain a wider perspective on the topic, we are going to conduct a survey study with a larger sample of respondents to investigate barriers and facilitators for engaging in implementation science to better understand how to promote implementation science in German-speaking countries. This will allow us to study differences in barriers and facilitators between various disciplines and to generate a more differentiated agenda for building a supportive implementation research infrastructure.

## Conclusion

Implementation research can have a highly positive impact on the availability of evidence-based interventions to the public if respective research projects are supported by the academic infrastructure. The present study suggests that establishing implementation science in German-speaking countries needs enhancements in several conditions, such as more training and funding opportunities. Simultaneously, implementation researchers should seize opportunities to advance their discipline, for example by advocacy and working toward a common understanding of what implementation science entails and offers. The results inform an agenda for the German-speaking implementation community and can be beneficial to other countries, especially those that are in the process of advancing their implementation research capacity and infrastructure.

## Supplementary Information

Below is the link to the electronic supplementary material.

Supplementary file1 (DOCX 19 kb)

Supplementary file2 (DOCX 17 kb)

Supplementary file3 (DOCX 34 kb)

## Data Availability

De-identified data from this study can be made available from the corresponding author on reasonable request.

## Code Availability

There is no analytic code associated with this study.

## Materials Availability

The materials used to conduct the study are available as supplementary files.
