# Supplementary material for: Barriers and Facilitators for Conducting Implementation Science in German-Speaking Countries: Findings from the Promote ImpSci Interview Study
Source: Glob Implement Res Appl. 2022 May 26;2(2):120–31. doi: 10.1007/s43477-022-00046-3 (PMC9134978; doi:10.1007/s43477-022-00046-3)
Supplement: Supplementary file 1 — Supplementary file1 (DOCX 19 kb) [file 43477_2022_46_MOESM1_ESM.docx]

**Supplementary File 1. SRQR Checklist**

Based on O’Brien, B. C., Harris, I. B., Beckman, T. J., Reed, D. A., & Cook, D. A. (2014). Standards for Reporting Qualitative Research: A Synthesis of Recommendations. *Academic Medicine*, *89*(9), 1245–1251. <https://doi.org/10.1097/ACM.0000000000000388>

*“Barriers and facilitators for conducting implementation science in German speaking countries:
Results of the Promote ImpSci interview study”, Global Implementation Research and Applications*

| **Reporting item** | **Page number** |
| --- | --- |
| Concise description of the nature and topic of the study identifying the study as qualitative or indicating the approach (e.g., ethnography, grounded theory) or data collection methods (e.g., interview, focus group) is recommended | 6-7 |
| Summary of the key elements of the study using the abstract format of the intended publication; typically includes background, purpose, methods, results, and conclusions | 1 |
| Description and significance of the problem / phenomenon studied: review of relevant theory and empirical work; problem statement | 2-6 |
| Purpose of the study and specific objectives or questions | 6 |
| Qualitative approach (e.g., ethnography, grounded theory, case study, phenomenology, narrative research) and guiding theory if appropriate; identifying the research paradigm (e.g., postpositivist, constructivist / interpretivist) is also recommended; rationale. The rationale should briefly discuss the justification for choosing that theory, approach, method, or technique rather than other options available; the assumptions and limitations implicit in those choices and how those choices influence study conclusions and transferability. As appropriate the rationale for several items might be discussed together | 7 |
| Researchers' characteristics that may influence the research, including personal attributes, qualifications / experience, relationship with participants, assumptions and / or presuppositions; potential or actual interaction between researchers' characteristics and the research questions, approach, methods, results and / or transferability | 7-8, 19 |
| Setting / site and salient contextual factors; rationale | 7 |
| How and why research participants, documents, or events were selected; criteria for deciding when no further sampling was necessary (e.g., sampling saturation); rationale | 6 |
| Documentation of approval by an appropriate ethics review board and participant consent, or explanation for lack thereof; other confidentiality and data security issues | 6-7 |
| Types of data collected; details of data collection procedures including (as appropriate) start and stop dates of data collection and analysis, iterative process, triangulation of sources / methods, and modification of procedures in response to evolving study findings; rationale | 7 |
| Description of instruments (e.g., interview guides, questionnaires) and devices (e.g., audio recorders) used for data collection; if / how the instruments(s) changed over the course of the study | 6-7 |
| Number and relevant characteristics of participants, documents, or events included in the study; level of participation (could be reported in results) | 8 |
| Methods for processing data prior to and during analysis, including transcription, data entry, data management and security, verification of data integrity, data coding, and anonymization / deidentification of excerpts | 7 |
| Process by which inferences, themes, etc. were identified and developed, including the researchers involved in data analysis; usually references a specific paradigm or approach; rationale | 7 |
| Techniques to enhance trustworthiness and credibility of data analysis (e.g., member checking, audit trail, triangulation); rationale | 7 |
| Main findings (e.g., interpretations, inferences, and themes); might include development of a theory or model, or integration with prior research or theory | 8-19 |
| Evidence (e.g., quotes, field notes, text excerpts, photographs) to substantiate analytic findings | 9-14 |
| Short summary of main findings; explanation of how findings and conclusions connect to, support, elaborate on, or challenge conclusions of earlier scholarship; discussion of scope of application / generalizability; identification of unique contributions(s) to scholarship in a discipline or field | 14-19 |
| Trustworthiness and limitations of findings | 19 |
| Potential sources of influence of perceived influence on study conduct and conclusions; how these were managed | Title page |
| Sources of funding and other support; role of funders in data collection, interpretation, and reporting | Title page |
