# Supplementary material for: Barriers and Facilitators for Conducting Implementation Science in German-Speaking Countries: Findings from the Promote ImpSci Interview Study
Source: Glob Implement Res Appl. 2022 May 26;2(2):120–31. doi: 10.1007/s43477-022-00046-3 (PMC9134978; doi:10.1007/s43477-022-00046-3)
Supplement: Supplementary file 2 — Supplementary file2 (DOCX 17 kb) [file 43477_2022_46_MOESM2_ESM.docx]

**Supplementary File 2. Topics of the Promote ImpSci interview study**

*“Barriers and facilitators for conducting implementation science in German speaking countries:
Results of the Promote ImpSci interview study”, Global Implementation Research and Applications*

**1. Experience with implementation science**

1.1 How did it come that you started working in the field of implementation research?

1.2 What are special characteristics of implementation science compared to other fields in your discipline?

1.3 How would you describe the status quo of implementation research in your field of work?

1.4 Do you see any differences in the status quo of implementation science in German-speaking countries compared to the international field?

**2. Facilitators & barriers for implementation science**

2.1 What have been important structural facilitators and resources for your own implementation research activities so far?

2.2 Which other facilitators and resources do you know in Austria/Germany/Switzerland or at the European level?

2.3 Are there any factors that you perceive as hindering for engaging in implementation research?

2.4 What research infrastructure would be needed to establish implementation science as a discipline in German-speaking countries?

2.5 Which other resources would be helpful?

**3. Resources and activities for German-speaking implementation researchers**

3.1 What are activities the German Speaking Implementation Association could offer to support the German speaking implementation community (this network currently provides a communication platform via LinkedIn and an email newsletter)?

3.2 As part of the Promote ImpSci project, an online workshop for implementation researchers in German-speaking countries will take place in March 2021. As a potential participant, what topics would you find relevant for such a workshop?

**4. Demographic data**

4.1 What is your current position at your research institution?

4.2 How long have you been working in the field of implementation science?

4.3 What has been the focus of your research in implementation science so far?
