# Supplementary material for: Barriers and Facilitators for Conducting Implementation Science in German-Speaking Countries: Findings from the Promote ImpSci Interview Study
Source: Glob Implement Res Appl. 2022 May 26;2(2):120–31. doi: 10.1007/s43477-022-00046-3 (PMC9134978; doi:10.1007/s43477-022-00046-3)
Supplement: Supplementary file 3 — Supplementary file3 (DOCX 34 kb) [file 43477_2022_46_MOESM3_ESM.docx]

**Supplementary File 3. Coding system of the Promote ImpSci interview study in the original German version and English translation**

*“Barriers and facilitators for conducting implementation science in German speaking countries: Results of the Promote ImpSci interview study”,
Global Implementation Research and Applications*

| **Thema 1: Charakteristika von Implementierungsforschung als Wissenschaftsdisziplin** | **Theme 1: Characteristics of implementation science as a scientific discipline** | **Examples** |
| --- | --- | --- |
| 1.1 Praxisorientierung von Implementierungsforschung | 1.1 Applied nature of implementation science | „You cannot just sit in a university and do implementation science from your desk. You need a field where you can really study it and learn. This applied aspect makes it difficult to separate clinical from academic aspects” |
| 1.2 Interdisziplinarität von Implementierungsforschung | 1.2 Interdisciplinarity of implementation science | “(…) it is characterized by interdisciplinarity, which makes it challenging, but also exciting” |
| 1.3 Abgrenzung von Implementierungsforschung | 1.3 Distinguishing implementation science | „I have the impression that a lot of things are understood as implementation science. There are examples of people, who think they conduct implementation science, because what they do is practice related. So, implementation science seems to be practice-related research to some people. (…) I think there is no common understanding about what implementation science is, at least not in (a German speaking country). For example, I ask myself if it is part of health services research or not.” |
| 1.4 Entwicklungsstand von Implementierungsforschung | 1.4 Developmental stage of implementation science | “In general, I see it as a relatively new field, especially in German speaking countries. In health services research, it is still a small subarea that is maybe gaining significance, perhaps also through new funding schemes in (a German speaking country), (…), maybe also when it comes to implementing new health services. It is constantly increasing, but I see it being rather in the beginning.” |
| 1.5 Sichtbarkeit von Implementierungsforschung | 1.5 Visibility of implementation science | “I don’t see anyone talking about implementation science in public, for example in relation to Covid-19, there are so many measures that are somehow decided upon and sometimes people talk about the need of (these measures) being evidence-based and effective. But nobody asks themselves if implementation science could be helpful in implementing these measures (…). In this regard, nobody ever thought about necessary preconditions or implementation strategies.” |
| 1.6 Internationale Unterschiede im Status Quo von Implementierungsforschung | 1.6 International differences in the status quo of implementation science | “This is also a phenomenon abroad, that you go ‘in and out’ of the field, so you conduct an implementation study, if its suitable, then you do something different, a clinical or sociological study. But there are also people who identify with implementation science in the long term. And that is missing in (a German speaking country).” |
| **Thema 2: Übliche Rahmenbedingungen für Implementierungsforschungsprojekte** | **Theme 2: Common conditions of implementation research projects** | **Examples** |
| 2.1 Dauer von Forschungsprojekten | 2.1 Duration of research projects | “You just need a long-term (perspective). For such projects you need other possibilities than just doing quick experiments for a year. And that is a structural barrier.” |
| 2.2 Kosten von Forschungsprojekten | 2.2 Costs of research projects | “I know from the US how expensive, how difficult, how involved implementation research is and I don’t see that there is money available for that.” |
| 2.3 Zusammenarbeit mit externen Einrichtungen | 2.3 Cooperation with external partner organizations | “It is extremely difficult to get resources, and therefore you need partners from practice and administration, who organize that and who have a better connection to policy makers, compared to scientists. (…) I couldn’t do it, I’m not a politician who negotiates things and acts strategically and thinks about how things look to the public. For that, you need people who do that and who are able to do that.” |
| **Thema 3: Individuelle Faktoren die mit dem Betreiben von Implementierungsforschung zusammenhängen** | **Theme 3: Personal factors that relate to conducting implementation research** | **Examples** |
| 3.1 Erkennen von Transferproblemen und -möglichkeiten | 3.1 Identifying transfer problems and possibilities | “(…) what I found so frustrating in my early research (…), that something was developed and largely evaluated, but then no one really did anything with it anymore” |
| 3.2 Nutzen von Gelegenheiten | 3.2 Making use of opportunities | “(…) the research group where I landed is pretty successful in raising research grants. Some of the projects can be described as implementation science. That is partly a coincidence, partly not.” |
| **Thema 4: Vernetzung von Implementierungsforscher*innen** | **Theme 4: Linkages with and networking among implementation scientists** | **Examples** |
| 4.1 Unterstützende Mentor*innen/Führungskräfte | 4.1 Supportive mentors/principal investigators | “(…) there is great support in our department because our management thinks it is important. If that would not be the case, someone like me would not be here and would not get any resources. That depends very much on specific people.” |
| 4.2 Unterstützendes Forschungsteam | 4.2 Supportive research team | “(…) supporting factors that I experience, are that I have the possibility of having people around me who are also interested in the topic.” |
| 4.3 Nationale Vernetzung | 4.3 National network | “(…) if I would write an implementation science research proposal in (a German speaking country), I would have to think hard about whom to include as a collaboration partner.” |
| 4.4 Internationale Vernetzung | 4.4 International network | „I think, I am in a special position here and I am very lucky, because I have such a good international network. If I wouldn’t have started that through European projects, I probably wouldn’t be interested in implementation science, because I wouldn’t even know about it.” |
| 4.5 Interdisziplinäre Vernetzung | 4.5 Interdisciplinary network | „If someone (in my discipline) would conduct implementation science, I think I had a chance to notice that. However, for example, if colleagues who are using (the same implementation instrument) in (a German speaking country) are from health services research (…). We couldn’t even meet at a conference; these are just different (research) areas.” |
| **Thema 5: Möglichkeiten des Erwerbs von Implementierungsforschungskompetenzen** | **Theme 5: Possibilities for acquiring implementation science competences** | **Examples** |
| 5.1 Selbstständige Aneignung von Wissen | 5.1 Independent acquisition of knowledge | “(…) I think it is quite difficult (to decide) where to begin – there is a variety of models and papers and frameworks and to really get into it – do I really have an overview? Sometimes I am not sure, if I got it right, even though I’ve been working on it for years and try to read a lot.” |
| 5.2 Formale Aus- und Weiterbildungsmöglichkeiten | 5.2 Formal education and training opportunities | “I think that implementation science is not a topic (in academic curricula) and because of that it is not known. (…) It would be good to get young people’s attention and to give them the opportunity of specific education and training.” |
| **Thema 6: Bereitschaft im Wissenschaftssystem, Implementierungsforschung zu etablieren** | **Theme 6: Readiness in academia for establishing implementation science** | **Examples** |
| 6.1 Einstellung zu Evidenzbasierung | 6.1 Attitudes toward evidence-based practice | “(…) there needs to be the assumption that evidence-based practice is a good idea. That it is possible to conduct studies that lead to results – not just one study but all studies together – that are the basis for recommendations or directions for practice or policy.” |
| 6.2 Bekanntheit von Implementierungsforschung | 6.2 General awareness of implementation science | “You still have to explain to a lot of people, also in academia, what (implementation science) actually is. Explain the difference between implementation and evaluation, generally this idea of implementation. I am part of various things in the political context that center around measures and there are consistent, incredible aha-moments that there are people who engage in this topic scientifically.” |
| 6.3 Bestehende Forschungsstrukturen an Hochschulen | 6.3 Existing academic structures | “I’m trying to advance implementation science and therefore, to acquire projects. On the one hand, I have good conditions, my position is secure (…). However, there is no further infrastructure, so no further positions, for example, to establish something. So, there are always isolated projects and that is my situation – I don’t think that it is different anywhere. If anything, worse.” |
| 6.4 Widmung von Stellen | 6.4 Establishing dedicated academic positions | “(…) it would need (…) one or more positions at the university that have the possibility of doing something like that on the long-term, without depending on short-term research projects. Someone who has knowledge in this field. To secure longevity and application, in order to be a contact person (…), something like a transfer position. (…) A transfer position is not the same as a professor in implementation science. But it would be important, the organizational interface to this kind of research and it would make it more visible and create a contact person and a reality.” |
| 6.5 Publikationsmöglichkeiten und -kultur | 6.5 Publishing opportunities and culture | “(…) you see that there are these editorial special issues on implementation science, and it shows the transversal influence of implementation science” |
| 6.6 Überzeugung von Stakeholdern im Wissenschaftssystem von der Relevanz von Implementierungsforschung | 6.6 Implementation science advocacy directed at stakeholders in academia | “Maybe we need to do more lobbying (for implementation science). To point out how important it really is. I don’t know (…) if it is known how long it takes for evidence-based interventions to come to practice, some of them never make it to practice and for many we know – I know a study that speaks about 17 years, and I think there are many more that have determined similar timeframes. I don’t know whether this is known among decision makers. Because then they wouldn’t just fund (intervention) development, development, development.” |
| **Thema 7: Finanzierung von Implementierungsforschung** | **Theme 7: Funding implementation science** | **Examples** |
| 7.1 Traditionelle Forschungsförderung | 7.1 Traditional research funding | “(A specific national funding agency) is an institution that primarily funds basic research and not applied research, but I also already got funding from (this agency) for applied research. It is not hopeless, and I think that the situation is somewhat better than it is the case in (another German speaking country). As far as I know, the counterpart (in the other country) only funds basic research and you won’t be funded as an applied researcher and what is left then? There are only ministries, the ministry of science or other ministries.” |
| 7.2 Implementierung als Begleitforschung | 7.2 Implementation science as accompanying research | “Something that I often criticize is that implementation science is seen as supporting research. That means that we (in our field) benefit from implementation research results, but our research does not advance implementation science as a field.” |
| 7.3 Vielversprechende Förderschienen | 7.3 Promising funding schemes | “Actually, I think that this idea of Horizon Europe would thematically open doors at some places to do something like that” |
| 7.4 Flexibilität im Einsatz von Forschungsmitteln | 7.4 Flexibility in the use of research funds | “(…) a certain degree of freedom with research funds, that I had, to do what I wanted. It didn’t go as far as structural support. Honestly, there were never dedicated resources, but the resource was, that there were people – and I wasn’t alone in that – who said, this is important, and we are given the freedom and possibility to do something for that.” |
| 7.5 Disziplinspezifische Forschungsförderung | 7.5 Discipline-specific research funding | “Ultimately, (project funding by a national funding agency) is the standard, that is what you hope to get. And if this is not possible, if implementation science is not funded for my discipline by (this agency) (…), if a proposal would be reviewed by traditional educational scientist, I don’t see any chance that such a project would be funded. I haven’t tried it, but that is my impression. I think that this is different if you work in health sciences (…).” |
